# Pulsed Laser Deposition of Epitaxial SrTiO_3_/Sr_3_Al_2_O_6_ Templates as a Water-Soluble Sacrificial Layer for GaAs Growth and Lift-Off

**DOI:** 10.1021/acs.cgd.3c01531

**Published:** 2024-08-28

**Authors:** Imran S. Khan, William E. McMahon, Chun-Sheng Jiang, Patrick Walker, Andriy Zakutayev, Andrew G. Norman

**Affiliations:** National Renewable Energy Laboratory, 15013 Denver West Parkway, Golden, Colorado 80401, United States

## Abstract

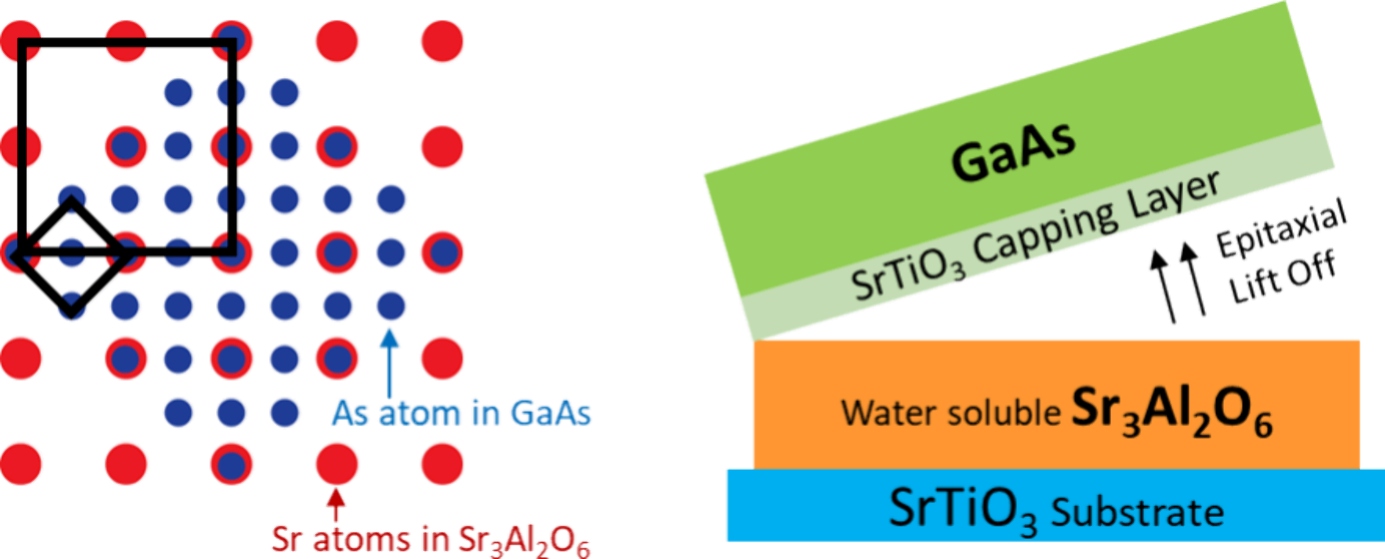

Despite the record-high efficiency of GaAs solar cells, their terrestrial application is limited due to both the particularly high costs related to the required single-crystal substrates and epitaxial growth. A water-soluble lift-off layer could reduce costs by avoiding the need for toxic and dangerous etchants, substrate repolishing, and expensive process steps. Sr_3_Al_2_O_6_ (SAO) is a water-soluble cubic oxide, and SrTiO_3_ (STO) is a perovskite oxide, where *a*_SAO_ ≈ 4 × *a*_STO_ ≈ (2√2)*a*_GaAs_. Here, the pulsed laser-deposited epitaxial growth of SrTiO_3_/Sr_3_Al_2_O_6_ templates on STO and Ge substrates for epitaxial GaAs growth was investigated, where SAO works as a sacrificial layer and STO protects the hygroscopic SAO during substrate transfer between deposition chambers. We identified that the SAO film quality is strongly dependent on the growth temperature and the O_2_ partial pressure, where either a high *T* or a high *P*(O_2_) improves the quality. XRD spectra of the films with optimized deposition parameters showed an epitaxial STO/SAO stack aligned to the STO (100) substrate, and TEM analysis revealed that the grown films were epitaxially crystalline throughout the thickness. The STO/SAO growth on Ge substrates at a high *T* with no intentional O_2_ flow resulted in some nonepitaxial grains and surface pits, likely due to partial Ge oxidation. GaAs was grown by metalorganic vapor-phase epitaxy (MOVPE) on STO/SAO/STO templates. Lift-off after dissolving the sacrificial SAO in water resulted in free-standing ⟨001⟩ preferentially oriented polycrystalline GaAs.

## Abstract 2

GaAs was epitaxially lifted off from a SrTiO_3_ substrate using Sr_3_Al_2_O_6_ as a water-soluble sacrificial layer, resulting in ⟨001⟩ preferentially oriented polycrystalline free-standing GaAs films.

## 1 Introduction

III–V solar cell technology enjoys a near monopoly for outer-space applications due to its high specific power and reliability. Single-junction and multijunction III–V solar cells exhibited record-high efficiency under 1 sun (global AM 1.5 spectrum).^1^ Yet the terrestrial application of GaAs solar cells is limited due to both the particularly high costs related to the required single-crystal substrates and epitaxial growth. Techno-economic analysis shows that approximately 84% of this cost is due to the use of expensive high-quality substrates.^2^ Therefore, a cost-effective substrate reuse technology can significantly bring down the total cost of the technology to enable widespread application.

The PV community has been heavily exploring different substrate reuse strategies such as epitaxial lift-off (ELO), mechanical spalling, and porous Ge release layers. However, the usefulness of all of the existing techniques is limited due to the need for toxic or harmful etchants, substrate repolishing, and/or expensive intermediate process steps. ELO is the most mature of the substrate reuse technologies, and proprietary techniques are already being used at small scale in the industry.^3^ The use of ELO for GaAs solar cell fabrication was demonstrated as early as 1978, where an AlGaAs sacrificial layer was selectively etched by using hydrofluoric acid. Since then, this method has been greatly improved^4^ and different techniques have been developed employing different sacrificial layers and etchant chemicals.^5,6^ Most of these chemicals are environmentally unfavorable. More importantly, high-quality GaAs growth on these recycled substrates after ELO becomes challenging due to surface roughness.^6^ Hence, there is a pressing need to develop new ecofriendly and cost-effective substrate removal and reuse techniques. A water-soluble lift-off layer could become just that by avoiding the aforementioned potential downsides. Other water-soluble lift-off layers are being explored such as NaCl and fluorides.^7−10^

**Figure 1:**
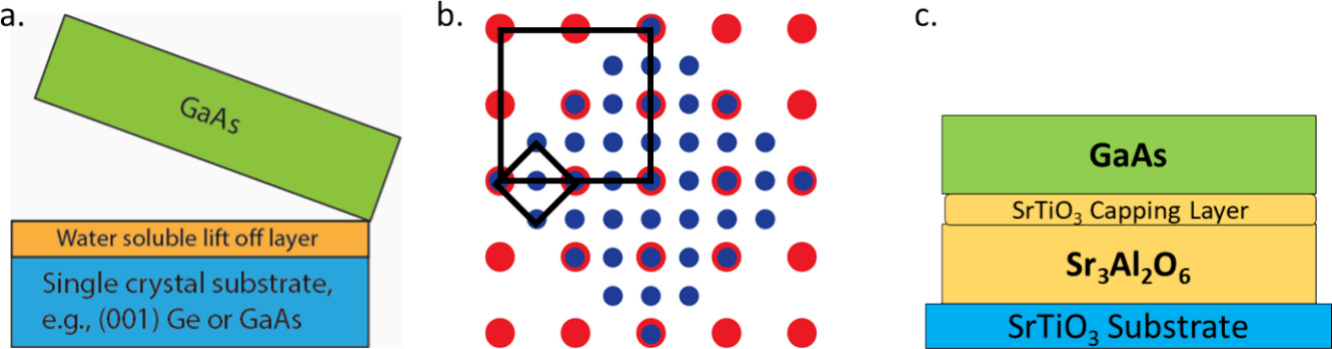
(a) Conceptual schematic for epitaxial GaAs lift-off using a water-soluble layer. (b) ⟨100⟩ SAO∥⟨110⟩ GaAs after a 45° lattice rotation. Blue represents As atoms in GaAs, and red represents Sr atoms in SAO. Black boxes outline the unit cells of the two crystals. (c) Material stack deposited in this study.

Sr_3_Al_2_O_6_ (SAO) is a hygroscopic cubic oxide that is highly water-soluble. Research interest in this material as a water-dissolvable lift-off layer has seen a recent increase—SAO has been demonstrated as a sacrificial buffer layer for ELO of perovskite oxides,^11−15^ Al_2_O_3_,^16^ and polycrystalline Ga_2_O_3_.^17^ Another attractive property of SAO for epitaxial buffer application is its mechanical flexibility, facilitating gradual strain control of the overlaying epitaxial film.^18,19^ SAO has a lattice constant of 1.5848 nm, which is close to (2√2)*a*_GaAs_ = 1.599 nm, giving a close lattice match between SAO ⟨100⟩ and GaAs ⟨110⟩ after a 45° lattice rotation (Figure 1). Due to the similarity of GaAs and Ge lattices, a similar relationship between Ge and SAO also holds. SrTiO_3_ (STO) is a perovskite oxide with a much smaller unit cell. However, in this case, 4 × *a*_STO_ = 1.562 nm, giving a lattice match between a single unit cell of SAO and four unit cells of STO. Four unit cells of STO can therefore coincidently lattice match with a 45° lattice rotated GaAs ⟨110⟩ (Table 1).

**Table 1:** Unit Cell Properties of the Relevant Material Crystals

material	crystal structure	space group	lattice constant, *a* (nm)	4 × *a*(nm)	2√2 × *a*(nm)
Sr_3_Al_2_O_6_	cubic	*P*a3̅	**1.5848**		
Ge	diamond	*Fd*3̅m	0.5657		**1.600**
SrTiO_3_	perovskite	*Pm3̅m*	0.3905	**1.562**	
GaAs	zincblende	*F*4̅3*m*	0.5653		**1.599**

Here, the epitaxial growth of SAO by pulsed laser deposition (PLD) and the GaAs growth by metalorganic vapor-phase epitaxy (MOVPE) were explored. Due to the required vacuum break and the extremely hygroscopic nature of SAO, a PLD-grown STO capping layer was deposited on top. We investigated these STO/SAO templates on GaAs, Ge, and STO substrates. Optimum growth conditions (substrate temperature and O_2_ partial pressure) for STO/SAO templates on STO substrates were identified based on X-ray diffraction (XRD) and transmission electron microscopy (TEM) data of the films. Growth on STO substrates was of superior epitaxial quality, and some degree of nonepitaxial grains was observed on Ge substrates. Hence, GaAs growth was attempted only on STO/SAO/STO templates. A substantial amount of epitaxially oriented GaAs (001) grains was observed for the GaAs films on these templates. Free-standing polycrystalline GaAs was demonstrated after lift-off. Optimization of the MOVPE deposition conditions and lift-off process may further improve the GaAs film quality.

## 2 Experimental Methods

The SAO and STO films were deposited inside a Neocera Combinatorial PLD System equipped with a Coherent COMPexPro 205 KrF excimer laser operating at 248 nm with a pulse duration of 10 ns. The laser, with an energy of 160 mJ and a repetition rate of 20 Hz, was focused with an area of 2.4 × 1.0 mm^2^ on a rotating 1 in. diameter commercial SAO or STO target (99.9% purity). The vacuum chamber base pressure was 4 × 10^–9^ Torr. The samples were mounted on a temperature-calibrated Inconel substrate holder heated with a resistive heater.

STO (001) substrates from MTI Corporation were rinsed with acetone and isopropanol. Right before loading in the deposition chamber, the STO substrates were held under running DI water for 1 min, followed by N_2_ blow dry. Prior to the thin film deposition, the substrate was annealed at 950 °C with 0.01 mTorr O_2_ for 30 min; this helped create an atomically flat titania terminated STO surface.^20^ The Ge (001) substrates from Umicore were cleaned by the following steps: NH_4_OH + H_2_O_2_ in a water solution dip, water rinse, HCl + H_2_O_2_ in water solution dip, water rinse, and finally N_2_ blow dry.

SAO was directly grown by PLD on STO or Ge substrates at different substrate temperatures and O_2_ partial pressures. The STO capping layer, also by PLD, was grown at fixed *T*_sub_ = 800 °C and an O_2_ partial pressure of 50 mTorr without breaking the vacuum. The crystallinity of the STO/SAO films was examined using a Rigaku SmartLab XRD instrument emitting Cu Kα radiation; the diffracted beam was probed through a 2-bounce Ge (220) monochromator.

GaAs was grown on an STO/SAO substrate in an atmospheric-pressure MOVPE reactor using arsine and triethylgallium sources. The growth rate was 6 μm/min and the V/III ratio of 30. The substrate was held at 650 °C, while 1.5 μm of GaAs was deposited.

TEM samples were prepared using standard lift-out techniques in a FEI Nova NanoLab 200 dual-beam focused ion beam (FIB) workstation using Ga^+^ ions. FIB damage was subsequently removed using low-energy (<1 kV) Ar^+^ ions, with the sample cooled using liquid nitrogen, in a Fischione model 1040 NanoMill. TEM was performed in either a FEI Tecnai SuperTwin TEM operated at 300 kV or a FEI Tecnai F20 UltraTwin field emitting gun (S)TEM operated at 200 kV. SEM energy-dispersive X-ray spectroscopy (EDX) and electron back scatter diffraction (EBSD) measurements were performed in a FEI Nova NanoLab200 FIB equipped with a Thermo Fisher Scientific UltraDry EDX detector and an Oxford Instruments Nordlys EBSD system, and a FEI Nova NanoSEM 630 SEM equipped with an Oxford Instruments Ultim Max EDX detector and Oxford Instruments Symmetry EBSD system.

Two experiments were performed to demonstrate ELO and the production of free-standing GaAs films. In the first, a piece of GaAs/STO/SAO/STO (substrate) sample was stuck GaAs growth surface down on Kapton tape and left in deionized water for 5 days at room temperature. The GaAs layer was lifted off the STO substrate using tweezers by peeling off the Kapton tape and GaAs together. This GaAs on Kapton tape was then bonded to the polished side of a (001) Si wafer with an EPO-TEK 353ND two-component epoxy and was cured at 170 °C for 10 min. The Kapton tape was peeled off with tweezers, leaving small areas of GaAs stuck to the Si wafer. The STO substrate was then heated in deionized water at 80 °C for 6 h to remove residual SAO as reported by Wang et al.^13^ The STO substrate and layer stack sample bonded to Si were then cleaned in acetone and methanol solvents at room temperature prior to further study. In the second, a piece of the GaAs/STO/SAO/STO(substrate) sample was bonded to the unpolished side of a (001) Si wafer using an EPO-TEK H20E two-component silver-filled conductive epoxy cured at 150 °C for 10 min. The sample was then left in deionized water for 4 days at room temperature to dissolve the SAO lift-off layer. The STO substrate was then removed using tweezers, and both the STO substrate and the GaAs/STO capping layer stack bonded to Si were heated in deionized water at 80 °C for 6 h to remove residual SAO. The substrate and bonded layer samples were then cleaned in acetone and methanol solvents at room temperature prior to further study.

## 3 Results and Discussion

### 3.1 SAO Growth on the STO (001) Substrate

**Figure 2:**
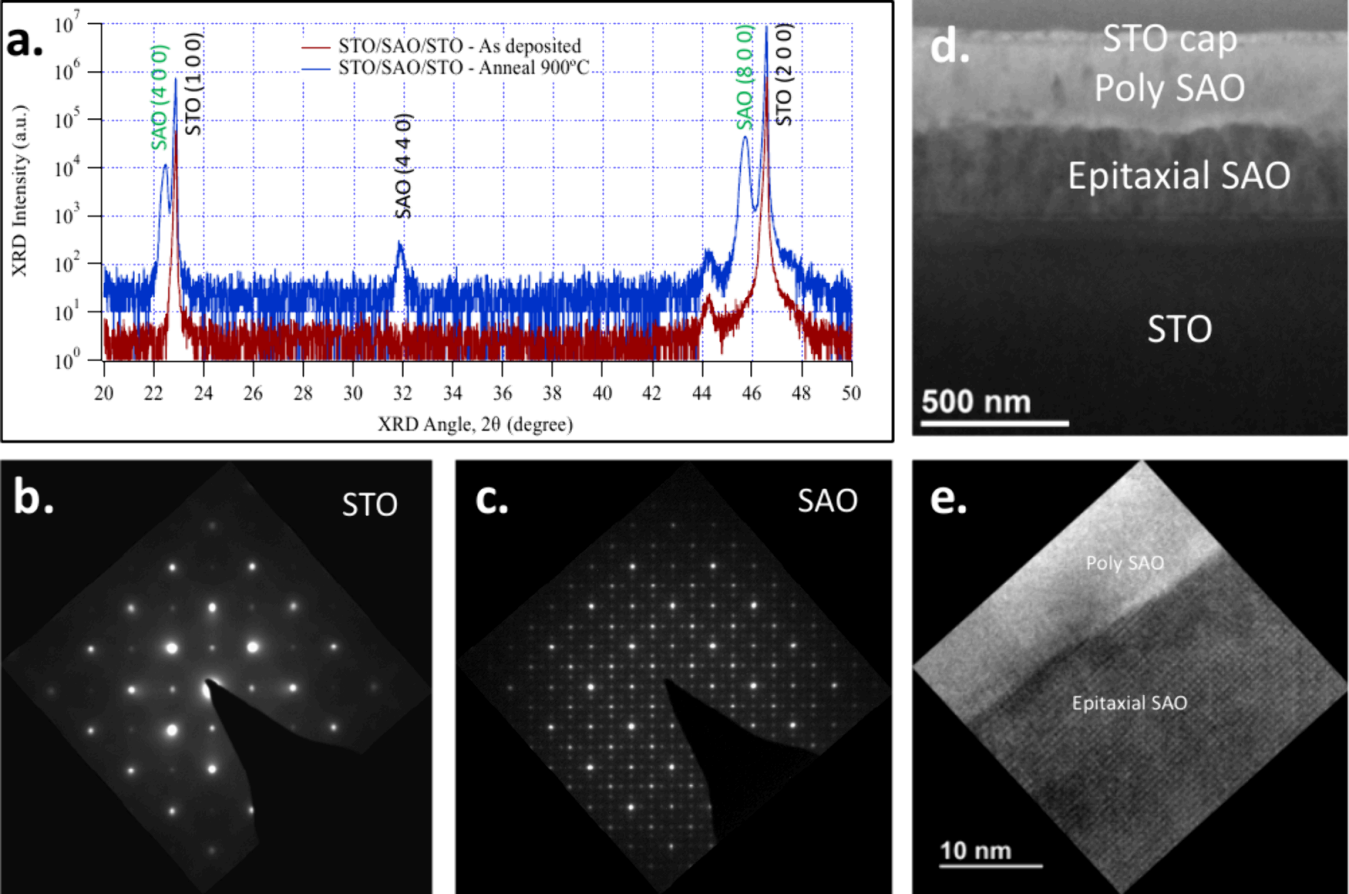
(a) XRD data of epitaxial SAO (100) on an STO (100) substrate before and after *ex situ* annealing. (b,c) TED patterns at the ⟨100⟩ pole of STO and SAO after annealing. (d) Bright-field TEM cross-section image of the STO/SAO/STO film and (e) HRTEM image of the SAO film.

For PLD growth of SAO on STO (001) substrates, the critical parameters for achieving epitaxial SAO were the O_2_ partial pressure and substrate set temperature (*T*_Sub_). SAO grew amorphously unless the right conditions were utilized. *Ex situ* annealing in an atmospheric air environment at 800 °C could epitaxially crystallize amorphous deposited SAO. XRD data showed that SAO (400) and SAO (800) peaks epitaxially aligned to the STO substrate (Figure 2a). However, an SAO (440) peak indicated that portions of the SAO thin film were not aligned to the substrate. TEM cross-section imaging showed that the film was not epitaxial throughout its thickness. The SAO layer closest to the STO interface was epitaxial after *ex situ* annealing, confirmed from the transmission electron diffraction (TED) pattern. The SAO film away from the substrate is possibly polycrystalline, containing the (440) orientation.

**Figure 3:**
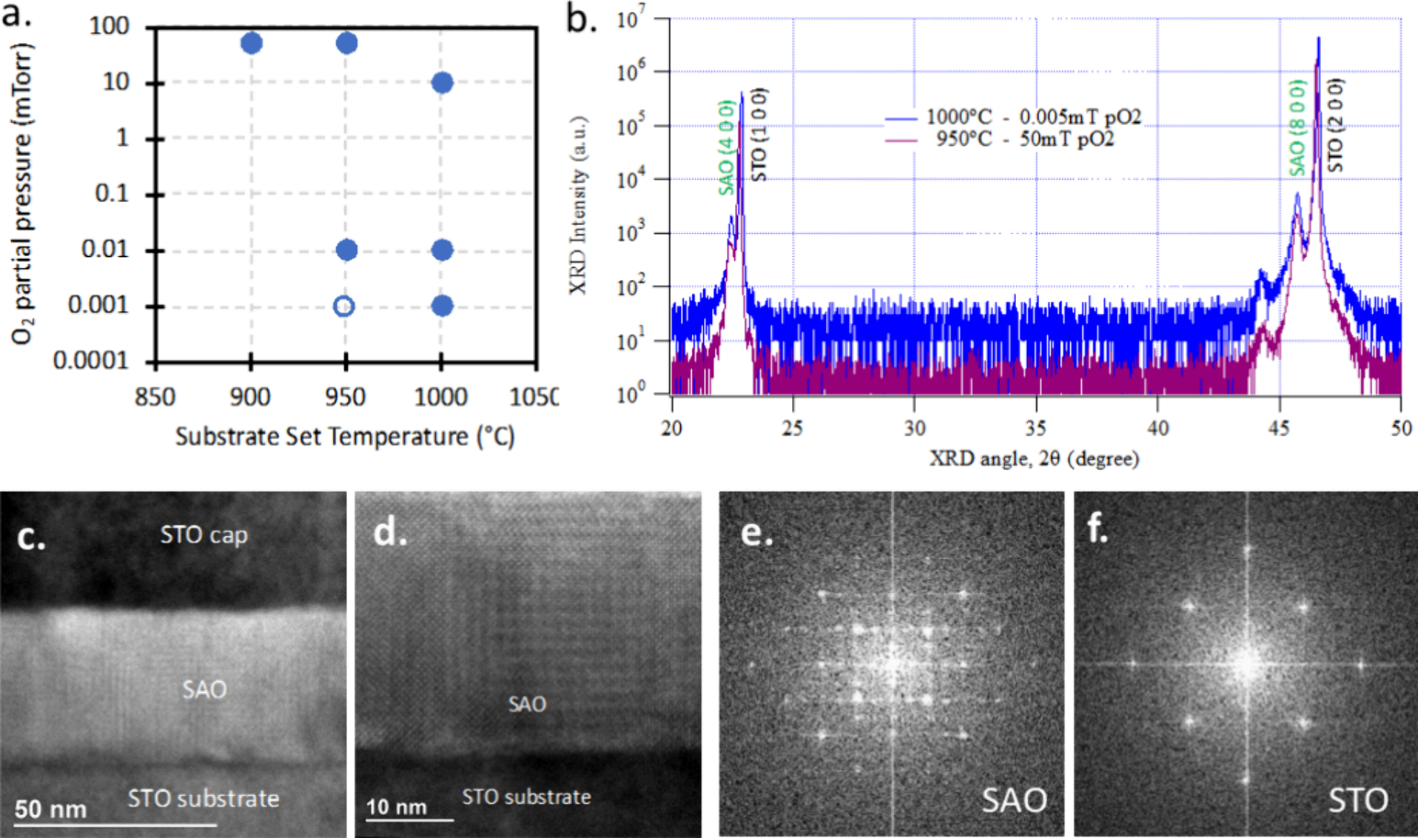
(a) Required *P*(O_2_) and *T*_Sub_ for epitaxial PLD growth of SAO. Blank circle indicates “partial” epitaxy. (b) XRD data for STO/SAO templates deposited on STO (001) substrates. (c) TEM and (d) HRTEM cross-section images of the STO/SAO/STO (substrate) template. Fast Fourier transform (FFT) of the (e) SAO film and (f) STO capping layer.

Direct PLD growth (no annealing) of epitaxial SAO on an STO substrate is possible by optimizing *T*_Sub_ and the O_2_ partial pressure. Figure 3a shows the deposition ambient pressure and temperature that resulted in epitaxially grown SAO. At higher *P*(O_2_), epitaxial growth was possible at lower *T*_sub_, lowering the temperature requirement to 900 °C. At the highest experimented *T*_sub_, epitaxial SAO could be grown without any active O_2_ flow; this could allow growing epitaxial SAO on substrates that are easily oxidized. XRD data (Figure 3b) indicated that epitaxial SAO peaks aligned to the STO substrates. No peaks related to nonepitaxial SAO or other phases were observed.

TEM data (Figure 3c–f) show that SAO growth is epitaxial throughout the thickness of the stack. Fast Fourier transform (FTT) of the high-resolution TEM (HRTEM) data revealed that the STO capping layer grown on SAO was also epitaxially aligned to the SAO layer (Figure 3e,f). The epitaxial growth of the STO capping layer is a qualitative indication of the high quality of the SAO surface. We also demonstrated the reuse of an STO substrate after dissolving off the STO/SAO for formation of a second-growth epitaxial STO/SAO template.

### 3.2 SAO Growth on the Ge (001) Substrate

**Figure 4:**
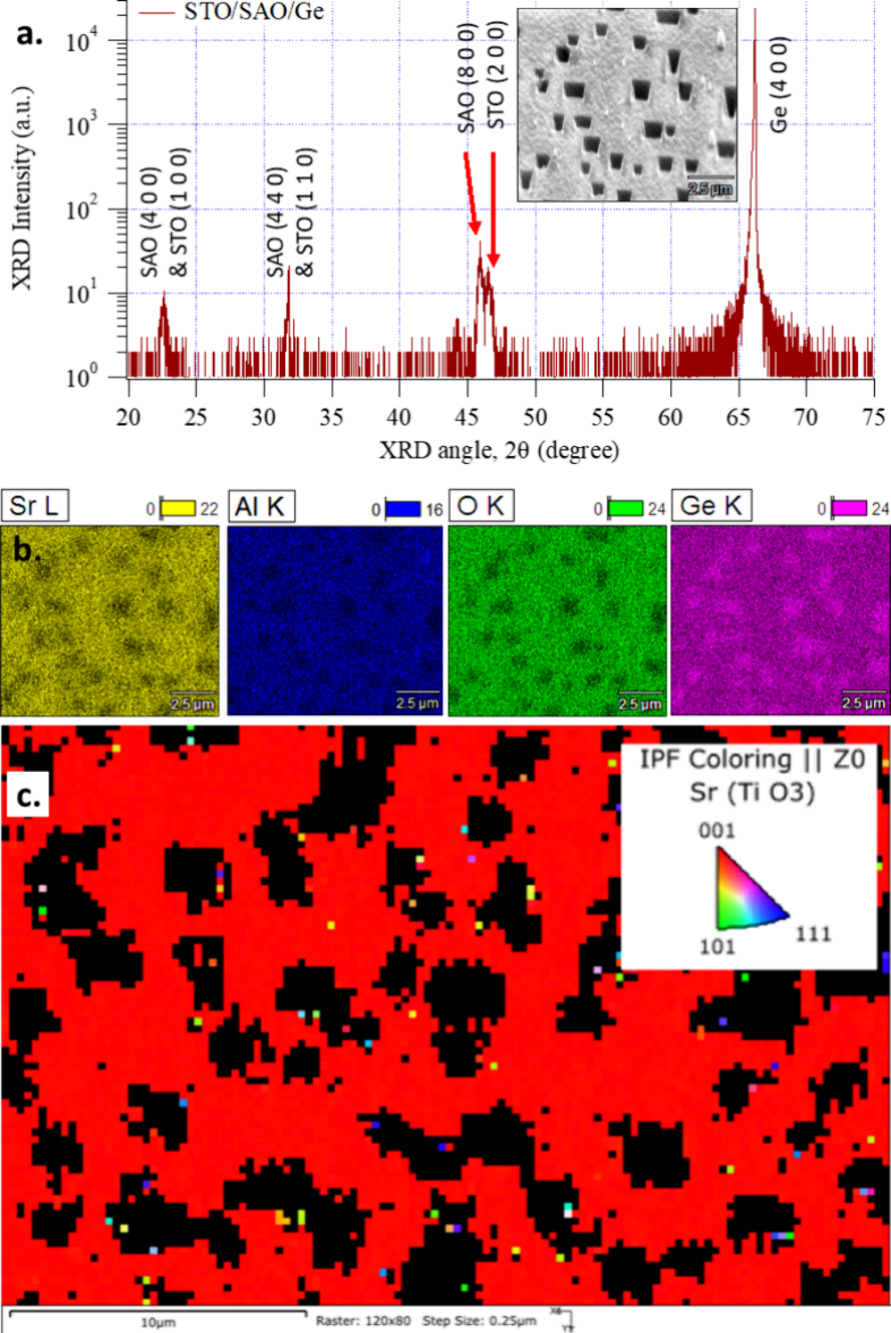
(a) XRD data for STO/SAO templates deposited on Ge (001) substrates. SEM plane view in the inset. (b) EDX elemental maps and (c) EBSD map of the STO/SAO/Ge templates.

The understanding of SAO growth on STO substrates was applied to growth on Ge (001) substrates. Deposition attempts with an O_2_ flow resulted in completely oxidized substrates. For SAO deposition at 1000 °C with *P*(O_2_) = approximately 5 × 10^–6^ Torr (no active O_2_ flow), XRD data showed epitaxial SAO and STO peaks along with nonepitaxial SAO (440) and/or STO (110) peaks (Figure 4a). This indicated that at least some regions of the deposited SAO film were epitaxially aligned to the Ge substrate and that allowed the growth of an epitaxially aligned STO capping layer.

SEM/EDX data (Figure 4b) showed a pitted surface for the STO/SAO/Ge (substrate) templates. These surface pits are possibly due to Ge oxidation, as EDX shows more Ge and less Sr, Al, and O in these pits. An EBSD map of the template (Figure 4c) revealed that STO and SAO between surface pits are epitaxial with some scatter in orientation. Further optimization of the deposition conditions may be possible to improve the STO/SAO/Ge template quality.

GaAs substrates could not withstand the required high temperature and O_2_ partial pressure for epitaxial quality SAO growth. Hence, STO/SAO growth results are reported only for growth on Ge and STO substrates.

### 3.3 GaAs Growth on STO/SAO Templates

**Figure 5:**
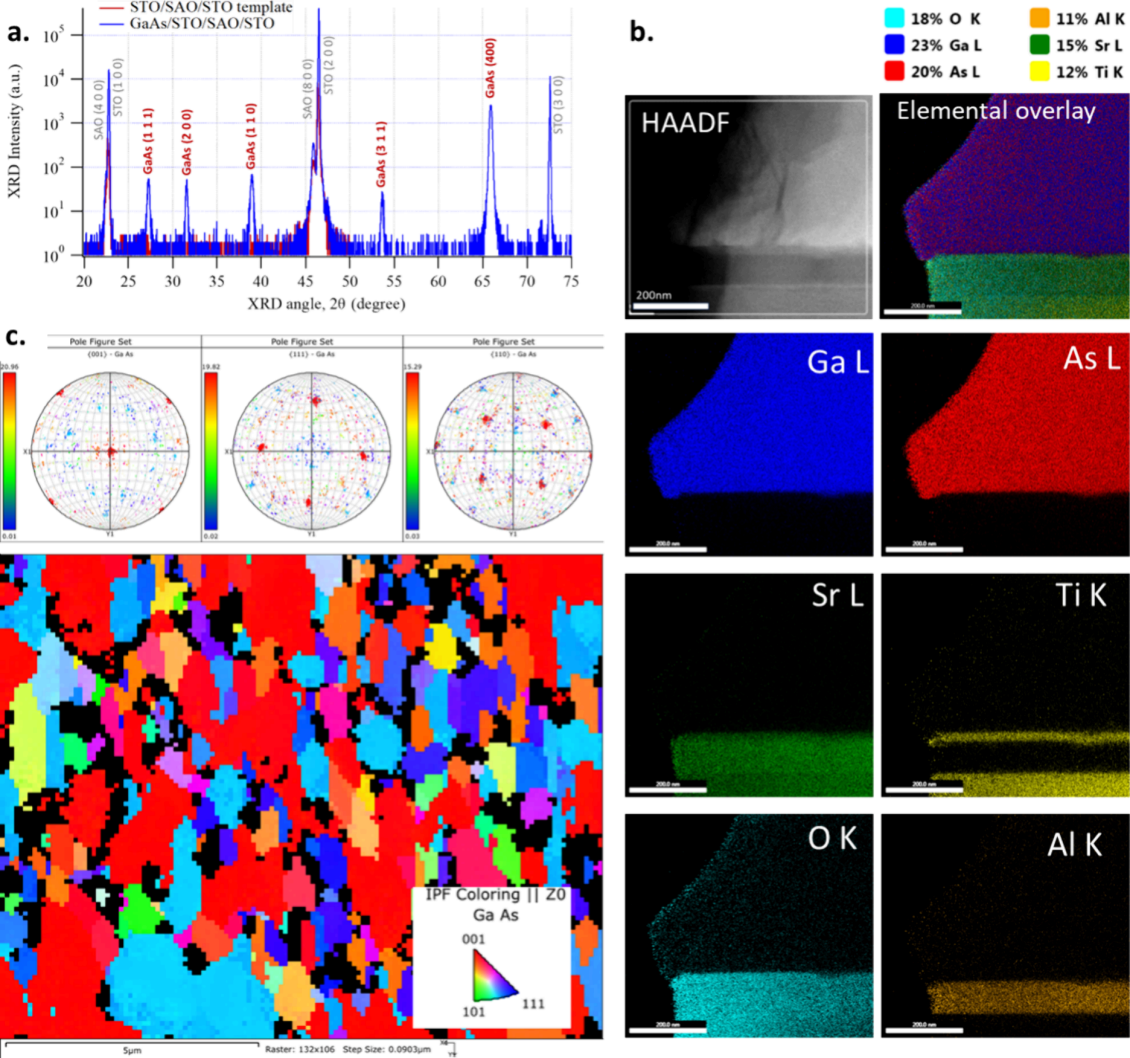
(a) XRD data for GaAs deposited on the STO/SAO/STO substrate template showing strong epitaxially oriented GaAs peaks. (b) Scanning TEM (STEM) high-angle annular dark field (HAADF) image and STEM EDX cross-section elemental maps of the GaAs/STO/SAO/STO (substrate) stack. (c) EBSD pole figures and map of the same sample.

GaAs with a thickness of approximately 1.5 μm was grown by atmospheric-pressure MOVPE on an STO/SAO/STO (substrate) template. XRD data indicated the presence of strong epitaxial GaAs (400) and (200) peaks along with nonepitaxial GaAs (110), (111), and (311) peaks (Figure 5a). The preferential orientation reported for polycrystalline GaAs grown with various deposition techniques and different substrates is (111), along with the presence of some (110) and (311) orientations.^21−23^ The GaAs films grown in this study having a preferential (100) orientation are a strong evidence that the growth is assisted by epitaxial alignment with the STO/SAO templates.

Scanning TEM (STEM) EDX cross-section maps (Figure 5b) of the GaAs/STO/SAO/STO (substrate) stack clearly show the layer structure. EBSD mapping (Figure 5c) of the sample indicates that a substantial amount of ⟨001⟩ grains in the GaAs is epitaxially oriented to the STO/SAO template (red areas in the EBSD maps). Analysis of the EBSD color map shows that approximately 40% of the area is occupied by epitaxial ⟨001⟩ grains, where ⟨111⟩ oriented grains occupied approximately 21% of the area.

**Figure 6:**
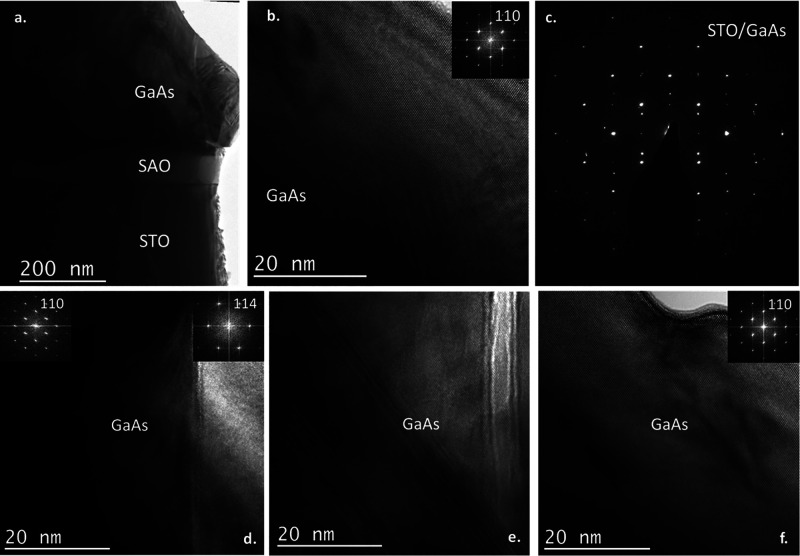
(a) Bright-field TEM image of the GaAs/STO/SAO/STO (substrate) stack. (b) Epitaxially aligned GaAs grain; FFT in the inset. (c) TED patterns showing the presence of epitaxially aligned GaAs grains in the GaAs layer. (d) Grain boundary between epitaxially aligned GaAs grains. Epitaxially aligned GaAs grains with (e) microtwins and (f) defects present in some areas.

Cross-sectional HRTEM analysis (Figure 6) also confirmed the presence of GaAs grains epitaxially aligned to the ⟨100⟩ STO/⟨100⟩ SAO/⟨100⟩ STO substrate stack. Some grains exhibited a ⟨110⟩ GaAs∥⟨100⟩ STO and (001) GaAs∥(001) STO epitaxial relationship while others exhibited a ⟨114⟩ GaAs∥⟨100⟩ STO and {221} GaAs∥(001) STO epitaxial relationship. GaAs grains also contain defects such as microtwins. Optimization of the MOVPE growth parameters could help achieve higher-quality GaAs films.

### 3.4 GaAs Lift-Off

**Figure 7:**
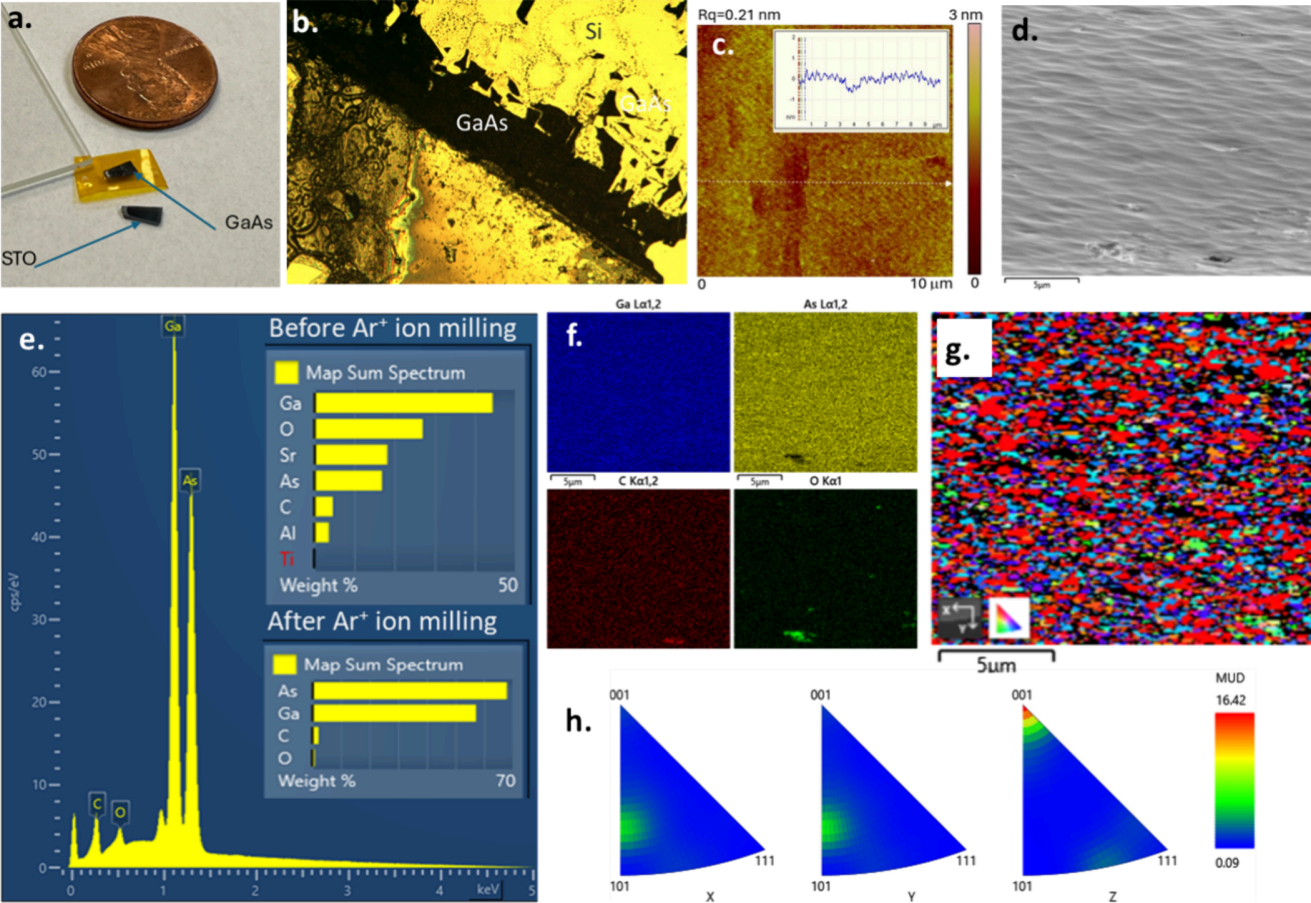
First experiment: (a) lifted-off free-standing GaAs/STO capping layer film from the GaAs/STO/SAO/STO substrate stack. (b) Optical image of lifted-off GaAs bonded to the Si substrate after transfer and removal of the Kapton tape. (c) Atomic force microscopy (AFM) measurement data of the STO substrate after GaAs lift-off. Second experiment: (d) SEM image, (e) EDX spectra, (f) EDX elemental maps, (g) EBSD IPF Z map, and (h) EBSD inverse pole figures of GaAs after Ar^+^ ion milling to remove the thin STO capping layer.

The GaAs and thin STO capping layer are successfully lifted off the GaAs/STO/SAO/STO substrate stack after bonding to either a piece of Kapton tape or to a Si wafer with conducting epoxy by dissolving the sacrificial SAO layer in water. The whole area of GaAs and STO capping layer was successfully transferred (GaAs growth surface down) to a Si wafer using conducting epoxy. For the sample bonded to Kapton tape, the GaAs film broke into smaller pieces during transfer and bonding to a Si wafer and the removal of the Kapton tape. It should be possible to optimize this lift-off process by the development of an improved bonding process to the Si wafer or other mechanically strong substrate. MOVPE growth optimization to obtain more perfect epitaxial GaAs films may also naturally result in more robust GaAs films for layer transfer. The remaining inert STO substrate surface after the lift-off and cleaning is very smooth as characterized by optical imaging and atomic force microscopy (Figure 7c). The AFM-measured root-mean-square roughness (Rq) values in the range of 0.20 to 0.32 nm are extremely promising according to literature^6^ and could likely be reused without any need for mechanical polishing. The samples of the lifted-off GaAs bonded to Si substrate with conducting epoxy were characterized with SEM-EDX and EBSD. Sr, Ti, O, and Al along with Ga and As elements were initially observed that were coming from the thin STO cap layer and some residual SAO (Figure 7e inset). After 5 kV Ar^+^ ion milling for 13 min to remove the STO capping layer, a more pristine GaAs surface was revealed (Figure 7d). SEM-EDX characterization now shows just Ga and As with only small amounts of C and O (Figure 7e,f). The EBSD analysis (Figure 7 g,h) shows polycrystalline GaAs with tendency for ⟨001⟩ oriented grains, similar to the observation on the top GaAs growth surface before lift-off.

## 4 Conclusions

The epitaxial growth of STO/SAO templates on STO (001) and Ge (001) substrates was demonstrated. The required growth temperature and O_2_ partial pressure for high-quality epitaxial SAO film were identified, demonstrating that either a high *T* or a high *P*(O_2_) could produce high-quality films. The templates on the STO substrates were of superior epitaxial quality through the thickness of the stack, while templates on the Ge substrate showed some nonepitaxial grains and surface pits. Initial GaAs growth by MOVPE on STO/SAO/STO (substrate) templates was promising. GaAs lift-off from the substrate stack was demonstrated after dissolving the sacrificial SAO layer in water. This resulted in ⟨001⟩ preferentially oriented polycrystalline free-standing GaAs films. Optimization of the MOVPE growth parameters and the lift-off process could result in higher-quality GaAs films.
